# Dairy and Fruit Listed as Main Ingredients Improve NRF8.3 Nutrient Density Scores of Children's Snacks

**DOI:** 10.3389/fnut.2020.00015

**Published:** 2020-03-10

**Authors:** Adam Drewnowski, Celine Richonnet

**Affiliations:** ^1^Center for Public Health Nutrition, University of Washington, Seattle, WA, United States; ^2^MOM Materne Mont Blanc Group, Paris, France

**Keywords:** nutrient profiling, nutrient-rich food index, ingredient list, dairy, fruit, snacks, WWEIA, food-based dietary guidelines

## Abstract

**Background:** The US Food and Drug Administration has modified its regulations on nutrient content claims by considering healthy dietary ingredients as well as nutrients.

**Objective:** To assess the relation between dairy and fruit as main ingredients in children's snacks and the Nutrient Rich Food (NRF8.3) nutrient density score.

**Methods:** Commonly consumed children's snacks in the United States, Canada, France, and the United Kingdom (*n* = 261) were assigned into USDA What We Eat in America (WWEI) categories. Nutrient composition data came from industry websites, open-source government databases (USDA Standard Reference SR28; CIQUAL), and back-of-pack food labels. Nutrient density was calculated using the Nutrient Rich Food Index NRF8.3. Snacks with dairy or fruit as the first listed ingredient (*n* = 115) were compared to those that listed neither (*n* = 146). Snacks that contained fruits-vegetables-nuts (FVN) (*n* = 88) were compared to those that did not (*n* = 173).

**Results:** NRF8.3 scores were higher for snacks listing dairy or fruit as main ingredients. Dairy or fruit when listed as the first ingredient were associated with higher percent daily values of protein, fiber, calcium, vitamin A, vitamin C, and vitamin D, lower saturated fat content and a 30-point increment in NRF8.3 scores. The presence of FVN was associated with a 22-point increment in NRF8.3 scores.

**Conclusion:** The correspondence between back-of-pack food ingredients and the nutrient based NRF8.3 scores suggests that ingredients can also be used to communicate the nutritional value of foods to the consumer. Dairy and fruit, when listed as first ingredients, were an important component of the NRF8.3 nutrient density score.

## Introduction

Methods to assess nutrient density of foods, based on their nutrient composition, have become known as nutrient profiling (NP) (1–6). Foods contain a variety of beneficial nutrients that may include protein, fiber, and a variety of required vitamins and minerals but they can also include excessive amounts of saturated fat, sugar, and salt (7). Designed to capture each food's overall nutritional value (2, 7). NP models aim to separate foods that are energy-dense from those that are nutrient-rich. Most NP models strive to include the beneficial nutrients to encourage and to limit excessive fat, sugar, and salt (4–7).

The development of NP models in the US and in the European Union (EU] has been guided by the regulatory environment (7–9). With some exceptions (6, 10), regulatory decisions on what foods qualify as “healthy” have been for the most part nutrient-based. The US Food and Drug Administration (FDA) standards required “healthy” foods to contain adequate amounts of protein, fiber, vitamin A, vitamin C, calcium, and iron (qualifying nutrients), without exceeding the limits for fat, saturated fat, cholesterol, and sodium (disqualifying nutrients) (1, 11). The initial NRF9.3 NP model in the US was accordingly based on protein, fiber, vitamin A, vitamin C, vitamin E, calcium, iron, potassium, and magnesium. It was recently modified to include vitamin D (1, 12). Nutrients to limit were saturated fat, added sugar, and sodium (8, 9).

In 2015, the food company KIND LLC submitted a citizen petition requesting that the FDA revisit its definition of a “healthy” food (13). This was in response to an FDA Warning Letter, which requested the company remove the word “healthy” from its packaging (14). KIND bars with nuts failed to meet the implicit nutrient content claim of “healthy” because they contained >1 g of saturated fats per Reference Amount Customarily Consumed (RACC) and because >15% of energy came from saturated fats. The KIND petition argued that purely nutrient-based standards were no longer supported by science and that the inclusion of healthy ingredients was more important than was the overall saturated fat content. The KIND petition asked the FDA to allow nutrient content claims on those products that contained meaningful amounts of health promoting foods, defined as vegetables, whole fruits, whole grains, legumes, and nuts. By May 2016, FDA reversed its earlier decision and allowed KIND to use the term “healthy” on its products.

This policy shift from healthy nutrients to healthy food ingredients was in line with current research on healthy food patterns in the US (15, 16). While the US Dietary Guidelines for Americans 2015–2020 have continued to stress the importance of excess saturated fat, sugar and salt, the focus of dietary advice has shifted toward the overall quality of habitual food patterns (17). Healthy food patterns are described as those with more low-fat dairy, more fruits and vegetables, and more whole grains, legumes, and nuts (17). Consistent with the US advice, the French National Plan for Nutrition and Health (PNNS) has recommended five servings of fruits and vegetables and three servings of milk and dairy per day (18).

The likely recognition of healthy food ingredients by US federal and other agencies opens the door to new hybrid NP approaches to NP modeling (1). While milk and fruit are viewed as desirable components of children's snacks, few studies have quantified the relation between ingredients listed on back-of-pack and algorithm-based nutrient density scores (19). The present approach was to screen a selection of commonly consumed children's snacks in the US, Canada, France, and the UK using a version of the Nutrient Rich Foods (NRF8.3) model. The contribution of milk and fruit listed as first ingredients on the back-of-pack ingredient label to NRF8.3 scores was examined as was the contribution to NRF8.3 scores of fruit, vegetables, and nuts.

## Materials and Methods

### Nutrient Composition of Snacks

Popular children's snacks (*n* = 261) in the US (*n* = 65), Canada (*n* = 60), France (*n* = 65), and the UK (*n* = 71) were identified through marketing research data in respective countries. The data were informed by sales figures from consumer research conducted by Nielsen (France, Canada, US), Ipsos (France, Canada), BCG (US), and Kantar (UK) and made available to MOM Materne Mont Blanc Group. The nutrient composition of snacks and the lists of ingredients were obtained from two main sources: (1) Back-of-pack nutrition fact panels and lists of ingredients, available from product packaging or from company websites; (2) government agency nutrient composition databases, some of them with data for branded products. The databases included the branded US Department of Agriculture Standard reference database SR28, now supplemented with a list ingredients for each product and the French nutrient composition database CIQUAL maintained by the French agency for food, environmental, and occupational health safety (ANSES), the French equivalent of the FDA. Nutrients missing from the nutrition facts panels or from company websites were imputed from nearest matches in the SR-28 for US and Canada and from the CIQUAL databases for France and the UK. Data for added sugar in US and Canada came from the USDA; data for added sugar in France came from a customized version of CIQUAL used in previous studies.

The 261 snacks were categorized by major categories and subcategories used in analyses of the What We Eat in America (WWEIA) data by the US Dietary Guidelines Advisory Committee 2015–2020 (US DGAC) (20). The major DGAC categories are dairy, protein foods, mixed dishes, grains, snacks and sweets, fruit, vegetables, beverages, and condiments (20). Not surprisingly, the typical children's snacks did not include vegetables or legumes, meat, poultry or fish, or eggs, mixed dishes, or condiments. Rather, most of the best-selling snacks came from such categories as sweet grains, candy and other sweets, dairy products (milk, yogurt, sugar sweetened beverages and 100% juices, fruits snacks, nuts, and seeds.

### The Nutrient Rich (NRF8.3) Model

The public-domain algorithm for the Nutrient Rich Food Index (NRFn.3) is given by NRn—LIM = NRFn.3, where n represents a variable number of nutrients to encourage and LIM represents 3 nutrients to limit (1–3). In published studies, the number of qualifying nutrients to encourage has varied from 6 (NRF 6.3) to 15 (NRF 15.3) (8, 9). The LIM score was always based on the same 3 nutrients of public health concern: saturated fat, added sugar, and sodium (8, 9). The basis of calculation was 100 kcal. The NRF score can be applied to foods, composite meals, and to total diets (12).

The present NR8 subscore was the sum of percentage daily values (%DVs) for 8 qualifying nutrients: protein, fiber, vitamins A, C, and D, calcium, iron, and potassium. Vitamin D replaced vitamin E and magnesium was omitted. The %DVs for qualifying nutrients were capped at 100%. The negative LIM sub-score was the sum of %MRVs (Maximum Recommended Values) for 3 disqualifying nutrients: saturated fat, added sugar, and sodium. Percent MRVs were also calculated for total sugar (21). Total sugars include those naturally present in fruit and/or dairy.

Nutrient density calculations are generally based on nutrient standards per reference amount of food, whether 100 kcal, 100 g, or serving size (11, 22, 23). Nutrient standards for the US and the European Union are close but not exactly the same. For ease of transnational comparisons, a single set of US-based standards was applied to snacks from all markets to assure uniformity in NP modeling.

The 8 nutrients to encourage and standard reference amounts were as follows: protein (50 g), fiber (28 g), vitamin A (800 μg), vitamin C (80 mg), vitamin D (15 μg), calcium (1,000 mg), iron (18 mg), and potassium (4,700 mg). The three nutrients to limit and maximum recommended values (MRVs) were: added sugar (50 g), saturated fat (20 g), and sodium (2,400 mg). The NRF8.3 was calculated as follows:

$$\begin{array}{r} NR8=\overset{8}{\underset{i=1}{\sum }}\frac{\frac{Content_{i}}{DVi}\times 100}{Energy\;density} \end{array}$$

and

$$\begin{array}{r} LIM=\overset{3}{\underset{i=1}{\sum }}\frac{\frac{Content_{i}}{MRV_{i}}\times 100}{Energy\;density} \end{array}$$

where content i is the food's content of each nutrient i, and DVi is the reference daily value (DV) for that nutrient. In NR calculation, each nutrient i is expressed in percentage of DV per 100 kcal (rather than per gram or serving). Percent DVs for nutrients to encourage were truncated at 100, so that an excessively high content of a single nutrient would not lead to excessive NRF scores.

### The Back-of-Pack Ingredient List

The USDA SR28 branded nutrient composition database, developed in collaboration with ILSI North America, includes both nutrients and ingredients (24). Ingredients are also provided on the back-of-pack label alongside the nutrition facts panel and can be obtained from company websites.

While no specific amounts are provided on the ingredients list, ingredients are typically listed in the order of importance. In the US, the section 101.4, Title 21 of the Code of Federal Regulation (CFR) (25) specifies how ingredients must be designated. The current rule of the US Food and Drug Administration is that the ingredients on a product label must be listed in order of predominance, with the ingredients used in the greatest amount first, followed in descending order by those in smaller amounts.

The number of back-of-pack ingredients can vary widely, depending on local regulatory requirements and the manufacturer's own clean label policy. For example, in the US, GoGoSqueez lists “apple, apple puree concentrate, lemon juice concentrate” whereas Haagen Dazs ice cream lists “cream, skim milk, egg yolks, vanilla extract.” KIND granola bar lists “oats, tapioca syrup, semi-sweet chocolate (unsweetened chocolate, cane sugar, cocoa butter, vanilla extract), honey, canola oil, brown rice, brown rice flour, cane sugar, sorghum, sea salt, quinoa, vanilla extract, vitamin E.” The ingredients of Oreo chocolate sandwich cookies are given as “sugar, unbleached enriched flour (wheat flour, niacin, reduced iron, thiamine mononitrate [vitamin B1], riboflavin [vitamin B2], folic acid), high oleic canola and/or palm and/or canola oil, cocoa (processed with alkali), high fructose corn syrup, leavening (baking soda and/or calcium phosphate), cornstarch, salt, soy lecithin, vanillin—an artificial flavor, chocolate.”

The more recent European Commission notice 2017/C393/05 has taken this a step further by providing guidelines on the quantitative declaration of ingredients (QUID) used in the manufacture of preprocessed foods (26). QUID is required when the ingredient (or category of ingredients) is included in the name of the food and is expressed as numerical percentage by weight. At this time, the QUID requirement covers products containing fruit or dairy (e.g., strawberry yogurt) and can be found on food labels in the EU. For example, strawberry flavored yogurt from Danone lists “whole milk (59.5%), skim milk powder, sugar (8.2%), red fruit (blackberry, strawberry, raspberry 5%), raspberry (5%), strawberry (5%).

The present analyses used two sets of criteria to examine the contribution of dairy or fruit to nutrient density. The presence of dairy was counted only if milk, yogurt, or cheese were listed as the first ingredient. Milk chocolate or milk powder, skim milk powder, or modified milk solids occurring further down the ingredient list did not qualify. The presence of fruit was counted only if fruit was listed as the first ingredient. Second place following water was allowed, but not after sugar or another ingredient Ingredients lists for e.g., orange juice from concentrate often take the form of: “water, oranges.” While fruit purees were included, cakes with fruit jam, fruit pectins or fruit flavors did not qualify. Following FDA position on added sugars, concentrated fruit juices (used as sweeteners in processed foods) did not count and neither did dairy components such as dehydrated milk solids.

Second, the products were classified by the presence of fruit, vegetables, or nuts (FVN), a more common approach, already used in some NP models (10). The FVN scoring system developed for the Food Standards Agency—Office of Communications (FSA-Ofcom) in the UK was initially based on recipes provided by the manufacturers (10). However, these procedures were recently modified by Public Health England (27). In the absence of recipes, we looked for the occurrence of FVN on the ingredient list. Peanuts and seeds were included in the FVN category. Vegetables were not a frequent ingredient of popular children's snacks. Starchy vegetables (potatoes, corn) in the form of chips did not qualify for inclusion in the vegetable category.

### Plan of Analysis

Nutrient content of snacks was expressed as % Daily Value (%DV) for nutrients to encourage and as % Maximum Recommended Value (%MRV) for nutrients to limit. Energy density (kcal/100 g), the NRF9.3 nutrient density scores and the LIM subscores were calculated for snacks aggregated by WWEIA categories. Snacks that contained fruit or dairy were compared to those that did not on both nutrients and nutrient density scores. Snacks that contained FVN were compared to those that did not. Tests for significant differences between means were based on one-way ANOVAs.

## Results

Energy density (kcal/100 g), the LIM subscores, and the NRF 8.3 scores for WWEIA categories are shown in Table 1. WWEIA identification codes and the number of items per category are provided as well. As expected, the most popular children's snacks in 4 countries were mostly cakes, cookies, brownies (*N* = 27), fruit candies (*N* = 27), chocolate candies (*N* = 19), sweet grains, and flavored yogurts. Mean energy density of children's snacks ranged from <50 kcal/100 g for whole fruit and fruit based soft drinks to more than 500 kcal/100 g for energy-dense chocolate, chips, and nuts. Among snacks with energy density <200 kcal/100 g were milk and yogurts, unsweetened fruit, 100% fruit juices, and other sweetened beverages. Among snacks with energy density of >200 kcal/100 g were fruit candy and snacks with added sugar, sweet grains, savory snacks, candy bars, and cheeses.

**Table 1:** Energy Density (ED) in kcal/100 g, LIM subscore, and NRF8.3 scores for snacks aggregated to WWEIA categories.

	**WWEIA food group**	***N***	**Energy density** **kcal/100 g**		**LIM subscore**		**NRF8.3 nutrient** **density score**		**WWEIA codes**
			**Mean**	**SD**	**Mean**	**SD**	**Mean**	**SD**	
1	Low fat milk, plain and flavored	6	59	22	16.09	6.16	37.67	24.48	1004, 1006, 1204, 1206
2	Cheese	13	293	114	22.95	10.31	14.38	15.99	1602
3	Yogurt, whole/reduced	21	96	26	23.38	8.10	25.49	29.30	1802
4	Yogurt, low fat/non-fat	18	80	18	18.20	8.26	30.25	18.59	1804
5	Nuts and seeds	16	504	97	14.28	9.89	2.72	13.99	2804
6	Muffins, biscuits	2	428	29	13.82	4.75	13.57	31.59	4402
7	Potato chips	6	534	22	8.60	2.97	1.98	9.60	5002
8	Corn chips	4	508	24	7.08	3.82	−1.93	3.94	5004, 5008
9	Popcorn	2	541	21	11.41	1.64	−0.62	7.24	5006
10	Crackers	10	463	36	11.59	3.84	0.66	8.11	5202, 5204
11	Cereal bars	15	390	35	11.49	6.72	22.06	21.03	5402
12	Nutrition bars	2	378	40	13.81	13.88	42.01	2.61	5404
13	Cakes, cookies, brownies	27	438	55	22.42	7.86	−9.53	11.46	5502, 5504, 5506
14	Candy, chocolate	19	517	34	32.02	8.16	−21.79	7.63	5702
15	Candy, fruit	27	331	68	12.67	13.83	22.38	44.44	5704
16	Ice cream	8	209	80	31.72	5.19	−11.79	11.39	5802
17	Pudding/dairy dessert	16	140	47	30.22	8.69	−2.62	17.09	5804
18	Ices	2	108	35	35.75	2.62	26.85	58.33	5806
19	Apples, bananas, berries	18	79	71	2.69	7.24	48.88	45.87	6002, 6004, 6010
20	Fruit, dried	2	316	23	0.59	0.29	19.58	2.76	6016
21	Fruit salad	7	70	8	10.08	15.86	40.62	36.78	6018
22	Citrus, apple, and other juices	7	48	2	0.33	0.26	67.55	24.11	7002, 7004, 7006
23	Sugar sweetened sodas	5	59	55	44.61	17.25	−43.98	16.94	7202
24	Fruit based soft drinks	8	40	10	30.46	18.51	−1.90	38.57	7204
**Total:**		261	277	185	18.63	13.40	12.26	33.87	

**Figure 1:**
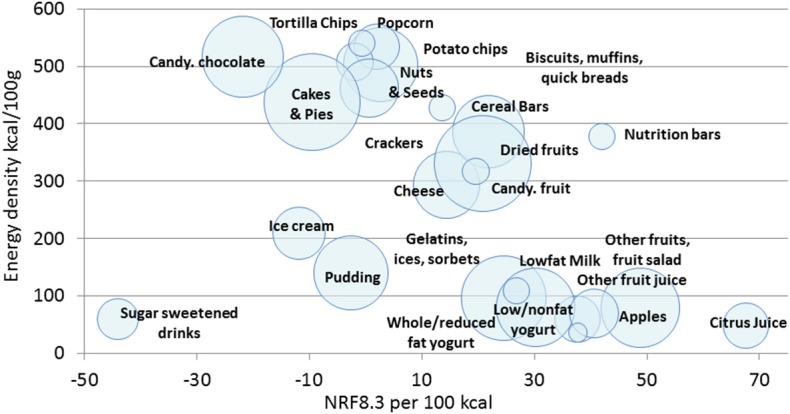
A scatterplot of NRF8.3 nutrient density per 100 kcal (x axis) and energy density kcal/100 g (y axis) of children's snacks the US, Canada, France and UK (*n* = 261). Snacks are aggregated by WWEIA food categories and labeled.

Figure 1 is a scatterplot of the relation between energy density (kcal/100 g) and nutrient density (NRF8.3) scores per 100 kcal for snack categories. Individual snacks were aggregated to WWEIA categories and the size of the circle denotes the number of items in each category. There was a clear separation between energy-dense snacks and snacks of lower energy density, which included unsweetened fruit and low-fat milk and yogurt. Chocolate candy, cakes, and crackers had relatively low NRF8.3 scores. Ice cream and pudding (dairy desserts) had lower energy density but also low NRF8.3 score, because of added sugar and saturated fat. Higher NRF8.3 scores were awarded to energy dense nutrition bars, cereal bars, dried fruit, cheese, and nuts and seeds.

**Figure 2:**
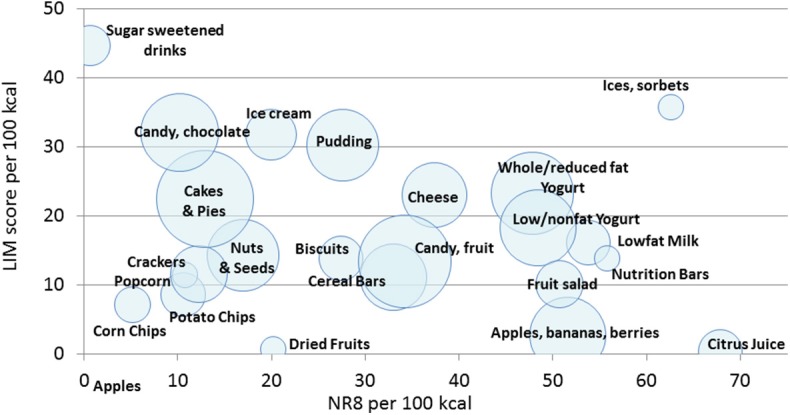
A scatterplot of NR8 nutrient density subscore per 100 kcal (x axis) and LIM scores (y axis) of children's snacks the US, Canada, France and UK (*n* = 261). Snacks are aggregated by WWEIA food categories and labeled.

Energy density (kcal/100 g) and LIM scores (saturated fat, added sugar and sodium) are correlated but not the same (8, 9). Figure 2 is a scatterplot of the relation between the LIM subscore and the nutrient density (NRF8) subscore, with snacks aggregated to WWEIA categories. There was an inverse relation between LIM subscores and NRF8.3 nutrient density score. The continuum of nutrient densities ran from sugar sweetened soft drinks to 100% citrus juice. Snacks based on milk, yogurt, and fruit had higher NRF scores as compared to candy, cakes and desserts.

**Figure 3:**
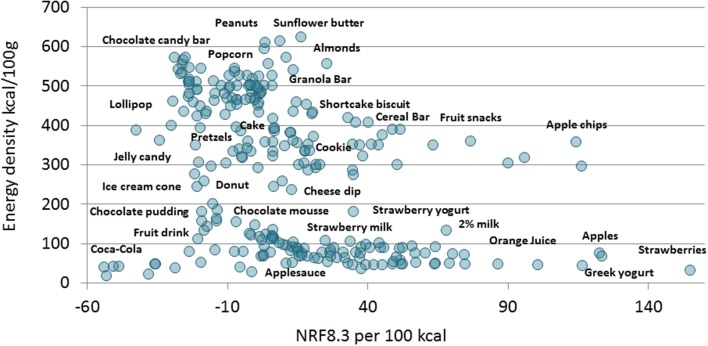
A scatterplot of NRF8.3 nutrient density per 100 kcal (x axis) and energy density kcal/100 g (y axis) of children's snacks the US, Canada, France and UK (*n* = 261). Snacks are shown individually and partly labeled.

Figure 3 is a scatterplot of the relation between energy density of individual snacks and their nutrient density (NRF8.3). The lowest nutrient density scores were for Coca-Cola; jellies and lollipops, chocolate candy, fruit drinks with added sugar and cookies and cake. The highest scores were for low-fat milk and low-fat plain yogurts, whole fruit, orange juice, Greek yogurt, and strawberries.

**Figure 4:**
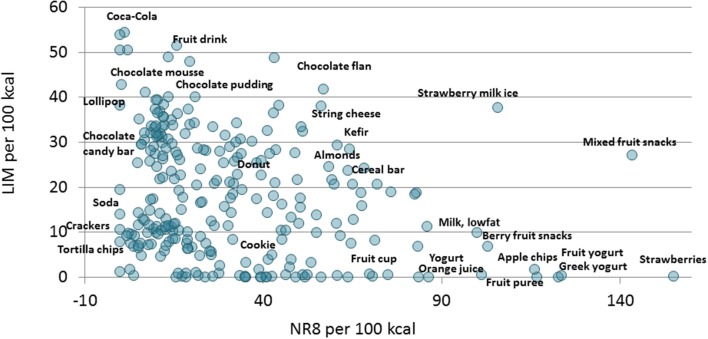
A scatterplot of NR8 nutrient density subscore per 100 kcal (x axis) and LIM scores (y axis) of children's snacks the US, Canada, France and UK (*n* = 261). Snacks are shown individually and partly labeled.

Again, energy density and LIM scores were not the same. Lower- energy density foods had variable LIM scores, depending on added sugar content. Figure 4 shows that the highest LIM and lowest nutrient density NR8 subscores were for a sugar sweetened beverage followed by other sweetened foods. By contrast, low-fat milk and low-fat yogurt scored higher as did apples, bananas and berries and citrus juice. Children's snacks based on unsweetened fruit, plain yogurt, and low-fat milk ranked relatively high on the nutrient density continuum.

### The Contribution of Dairy and Fruit as Main Ingredients to NRF8.3 Scores

The relation between dairy or fruit, listed as first ingredients on back-of-pack and the NRF8.3 score is shown in Table 2. Also shown are percent daily values (%DV) for NRF8.3 components and the NR8 and LIM subscores. When milk, yogurt, cheese or fruit were listed as the first ingredient, the snacks were higher in protein, fiber, vitamin A, vitamin C, vitamin D, calcium, iron, and potassium. Total sugar was higher because of naturally occurring sugar in milk and fruit; there was no difference in saturated fat, added sugar, or sodium. As might be expected, higher protein, vitamin A, vitamin D, calcium, and potassium came from dairy, whereas higher values for fiber and vitamin C were due to fruit.

**Table 2:** Nutrient composition %DV 100 kcal of children's snacks by the first ingredient listed on the ingredient label.

	**Fruit or Dairy** ***n* = 115**	**Neither** ***n* = 146**		**Fruit vegetables** **nuts *n* = 88**	**No. fruit vegetables** **nuts *n* = 173**	
	**Mean (SEM)**	**Mean (SEM)**	***P***	**Mean (SEM)**	**Mean (SEM)**	***P***
%Daily Value/100 Kcal						
Protein	6.13 (0.46)	2.71 (0.18)	<0.001	4.03 (0.43)	4.31 (0.31)	0.587
Fiber	3.63 (0.58)	2.39 (0.28)	<0.05	5.04 (0.60)	1.86 (0.30)	<0.001
Vitamin A	5.48 (0.80)	1.15 (0.43)	<0.001	2.81 (0.74)	3.17 (0.56)	0.697
Vitamin C	12.14 (2.30)	6.16 (1.50)	<0.05	15.98 (2.90)	5.13 (1.27)	<0.001
Vitamin D	3.03 (0.48)	0.31 (0.11)	<0.001	1.66 (0.42)	1.35 (0.26)	0.544
Calcium	10.40 (1.08)	2.44 (0.35)	<0.000	5.30 (1.26)	6.27 (0.57)	0.419
Iron	1.47 (0.33)	3.08 (0.32)	<0.001	2.16 (0.44)	2.49 (0.28)	0.522
Potassium	3.35 (0.22)	0.99 (0.10)	<0.001	2.96 (0.26)	1.55 (0.14)	<0.001
%Maximum Recommended Value/100 Kcal						
Saturated fat	5.29 (0.64)	6.00 (0.47)	0.365	3.42 (0.47)	6.84 (0.51)	<0.001
Total sugar	16.28 (0.76)	10.62 (0.65)	<0.001	16.89 (0.94)	11.19 (0.58)	<0.001
Added sugar	9.25 (0.92)	11.66 (1.10)	0.105	8.34 (1.06)	11.67 (0.96)	<0.05
Sodium	2.06 (0.21)	2.53 (0.18)	0.095	1.21 (0.13)	2.89 (0.19)	<0.001
ED (kcal/100 g)	126 (9)	395 (12)	<0.001	213 (20)	309 (13)	<0.001
NR8 subscore	45.65 (2.46)	19.12 (1.80)	<0.001	40.00 (3.30)	26.16 (1.82)	<0.001
LIM subscore	16.60 (1.20)	20.19 (1.11)	<0.05	12.97 (1.35)	21.49 (0.97)	<0.001
NRF8.3 score	29.05 (3.01)	−1.07 (2.25)	<0.001	26.99 (3.93)	4.68 (2.14)	<0.001

Those snacks that listed dairy or fruit as the first ingredient had higher NRF8.3 scores compared to those that did not. Energy density was significantly lower (126 kcal/100 g compared to 395 kcal/100 g). The NR8 score was higher and the LIM subscore was lower. The NRF8.3 score was higher by an average of 30 points (*p* < 0.001).

Those snacks that listed FVN also had higher NRF8.3 scores compared to those that did not. Fewer children's snacks contained vegetables and so the category size was reduced. As expected, listed FVN content was associated with higher %DV values for fiber, vitamin C and potassium (but not protein). Saturated fat was reduced, total sugar was increased (fruit) and there was a reduction in added sugar and sodium. Energy density was significantly lower (213 kcal/100 g compared to 309 kcal/100 g). The NR8 score was higher and the LIM subscore was lower. The NRF8.3 score was higher by an average of 22 points (*p* < 0001).

## Discussion

The present analyses were based on branded open-source nutrient composition databases for commonly consumed children's snacks in the United States, Canada, France and the United Kingdom, as identified by marketing research. Energy density and nutrient density measures were obtained. Energy density of children's snacks has been used in the past to distinguish between healthier and less healthy snacks (28). The present snacks varied greatly by energy density per 100 g. Energy-dense snacks (>200 kcal/100 g) were chocolate, nuts, chips, cakes, cookies and brownies and other sweetened grains. Lower energy density snacks (with energy density <200 kcal/100 g) were fruits and juices, milks, and plain and flavored yogurts.

Energy density per 100 g is a measure of policy interest. In January 2014, Mexico passed an 8% tax on non-essential foods—that is snacks—with energy density ≥275 kcal/100 g. The tax was imposed on salty snacks, chips, cakes, pastries, and frozen desserts; and a 1 peso/liter (~10%) tax on sugar-sweetened beverages. Basing the tax on energy density rather than on healthy ingredients or nutrient content meant that nuts and cereal bars were taxed whereas ice cream was not. By contrast, some past evaluations of nutrient density of snacks, some based on the NRF nutrient density score (29), have focused more on the snacks' nutritional value rather than on their energy density alone. When it comes to snacks, dietary advice has focused on promoting snacks of lower energy density and higher nutrition value (29–31).

It should be noted that some energy-dense snacks were relatively nutrient rich (32); for example, nuts and fortified cereals both had high NRF8.3 scores. By contrast, some low energy-density beverages were nutrient-poor (soda and fruit drinks). As shown above, the LIM score was distinct from energy density since it penalized added sugar in low energy density beverages. In the present dataset, about 60% of the snacks contained added sugar. Although the mean %DV for added sugar was 10%, sugar sweetened beverages and sugar candy were essentially all sugar, providing 100% of energy from added sugar.

Examining the contribution of first-listed ingredients to NRF8.3 scores is a novel component of this paper. This approach follows on the Code of Federal Regulations in the US and the more recent 2017 notice by the European Commission. In the US, the ingredients on a product label must be listed in the order of predominance, with the ingredients listed in the greatest amount listed first. The European Commission has imposed quantitative requirements for key ingredients to be listed as numerical percentage by weight. The implementation of these requirements will open the door to a new generation of NP models that make use of the ingredient list. In the present analyses, dairy or fruit listed as the first ingredient were associated with a 30-point increase in the NRF8.3 nutrient density score. The presence of FVN produced comparable results but the differences were less sharp.

While most NP models are based on nutrients only, some do award additional points to selected food groups, notably FVN. For example, the FSA-Ofcom guidelines (10) and the Australian Health Star Rating System (33) award extra points to foods containing fruit, vegetables, and nuts. The French Nutri-Score (34), directly derived from FSA-Ofcom, has awarded extra points to foods containing fruit, vegetables, legumes, nuts or rapeseed, and to olive or nut oils.

However, the relation between the foods' content of FVN and the nutrient profiling algorithm has always seemed arbitrary. The algorithm used to assess the amounts of fruits, vegetables, or nuts in complex foods for the FSA-Ofcom score was particularly complex (10). The FVN content of foods was then rated along a wholly arbitrary range of 5 points, based on the percent content of FVN per 100 g of food product. Only products that contained >80% FVN were awarded 5 points; products that contained <40% FVN got 0 points, those that contained >40% got 1 point and those that contained >60% got 2 points. The nutrient profiling technical guidance published in 2011 by the Obesity Team at the UK Department of Health runs to 18 detailed pages (10). It is a summary of a more extensive document that is no longer available online (35).

The principal consideration was that only intact fruit and vegetables (including those that were cooked and dried) and those that were minimally processed (peeled, sliced, tinned, frozen, 100% juices, and purees) could be included when calculating the FSA-Ofcom score. More detailed instructions on how to calculate the FVN content of foods for an updated UK nutrient profiling model were recently published by Public Health England (27). Neither document was meant to assist the shopper. Rather, the PHE guidelines had been produced to assist food manufacturers, retailers and advertisers to correctly calculate nutrient profiling scores for their products (10, 27). That required manufacturers to provide the weight of each ingredient in a product, which would then allow an exact calculation of the fruit, vegetable, and nut content in a food. The present approach was to base calculations on the ingredient listed first, consistent with the US and the EU guidelines. Following the FDA practice, concentrated fruit juice sugars, powders, or leathers were not counted as fruit in the present study. The nut category included peanuts as well as tree nuts.

There are other studies suggesting that the back of pack ingredient list is a potential additional tool for nutrition education. Lacking detailed information on the weight of FVN in food products, the Environmental Working Group in Washington DC (36) developed a separate algorithm that used the order of the ingredient's listing as a proxy for the percentage of fruit, vegetable, or nut in the product. The nutrient content of FVN ingredients was then compared to the foods' content of carbohydrates, sugar or fat in a ratio-based metric (36). The present approach was to quantify the contribution of dairy or fruit listed as first ingredients to the previously established NRF8.3 score. On average, snacks with dairy or fruit as first ingredients scored 30 points higher. Arguably, the inclusion of dairy is more relevant to the rating of children's snacks than are vegetables, which were barely represented in the present market-driven database.

As the dietary guidance is shifting from nutrients to whole foods and food ingredients, NP models need to follow suit. The 2015–2020 DGA defined healthy dietary patterns as composed of a variety of vegetables from all of the subgroups; fruits; grains, at least half of which are whole grains; fat-free or low-fat dairy; a variety of protein foods; and oils (1). Hybrid NP models that combine both nutrients and selected dietary ingredients are more closely aligned with dietary guidance that is increasingly targeted at foods and food groups, rather than isolated nutrients. Some hybrid scores have attempted to include whole grains, plant proteins, seafood, or healthy oils, consistent with the DGAs. However, some of those food groups seem to be underrepresented in children's snacks—hence the resent focus on dairy and fruit, in comparison to a modified FVN approach.

Children's snacks are not often viewed as particularly nutritious and many fall into the category of processed or “ultra-processed foods” (37–39). Whereas, NP models were initially intended to guide consumer behaviors at the point of sale, the NP methodology is increasingly being used by food companies for product screening and reformulation. Those voluntary efforts are monitored by the Access to Nutrition Index (ATNI), a global initiative to evaluate the world's largest food and beverage manufacturers on their policies and practices, including the nutritional value of their product portfolios (40).

## Conclusions

Dairy or fruit, when listed as first ingredients, made an important contribution to the NRF8.3 nutrient density score. The correspondence between nutrient based NP models and the back-of-pack food ingredients suggests that ingredients can also be used to communicate the nutritional value of foods to the consumer. Data from the ingredient list, increasingly available in electronic format, can also be used to construct a new generation of hybrid NP scores for potential use in the (re) formulation and optimization of food products.

## Data Availability Statement

The US Department of Agriculture Branded Food Products database is available at FoodData Central: https://fdc.nal.usda.gov/. The French food composition table CIQUAL is available at https://ciqual.anses.fr/. Additional data came from manufacturers' websites and food labels.

## Author Contributions

AD and CR conceptualized the study and reviewed and approved the manuscript. CR generated the list of snacks from multiple markets and provided nutrient composition data. AD conducted nutrient profiling analyses and took the lead on writing the manuscript. The views expressed in this work are those of the authors and do not necessarily reflect the position or policy of MOM Materne Mont Blanc Group.

### Conflict of Interest

The authors declare that this study received funding from MOM Materne Mont Blanc Group. CR is the Nutrition Director for MOM Materne Mont Blanc Group. CR was involved in conceptualizing study design, identifying snacks for nutrient profiling, and identifying open source websites with nutrition information. The funder was not involved in data collection, data analysis, interpretation of data, or the writing of the initial draft of the article. Both authors took the decision to submit the manuscript for publication. AD, the originator of the NRF family of nutrient density scores, has received grants, contracts, honoraria, and consulting fees from numerous food and beverage companies and other commercial and nonprofit entities with interest in nutrient profiling of foods.
